# Supplementary material for: Neurogenetic profiles delineate large-scale connectivity dynamics of the human brain
Source: Nat Commun. 2018 Sep 24;9:3876. doi: 10.1038/s41467-018-06346-3 (PMC6155203; doi:10.1038/s41467-018-06346-3)
Supplement: Supplementary file 1 — Supplementary Information [file 41467_2018_6346_MOESM1_ESM.docx]

***Neurogenetic Profiles Delineate Large-scale Connectivity Dynamics of the Human Brain***

Ibai Diez and Jorge Sepulcre

**
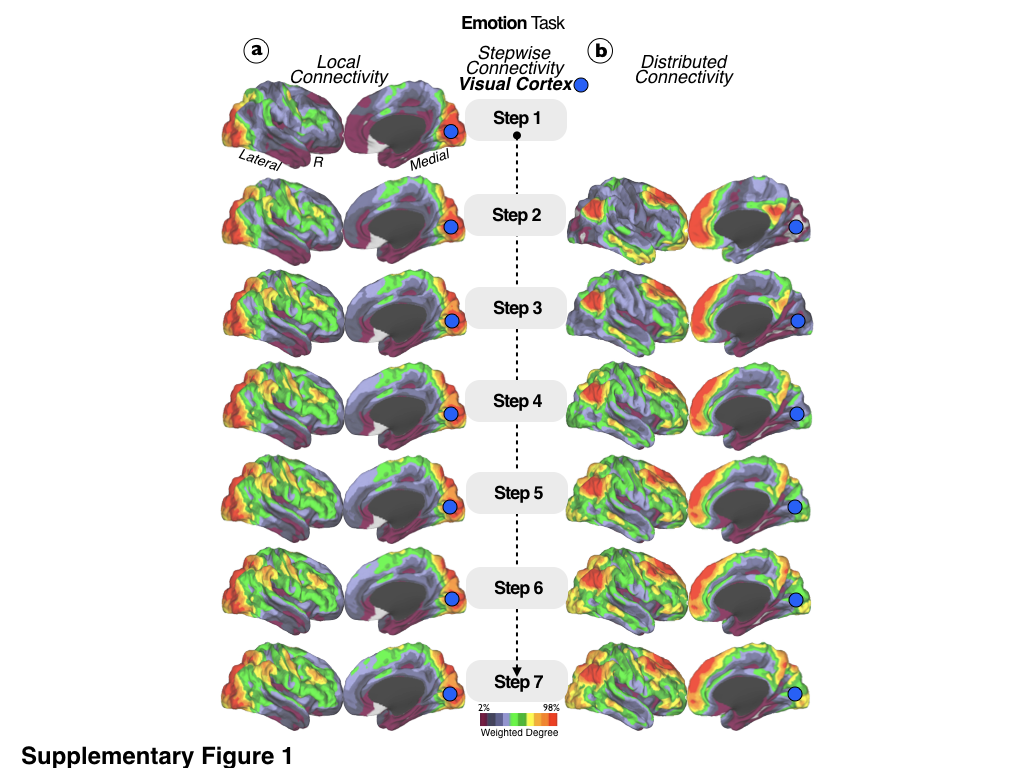
**Supplementary Figures:

Supplementary **Figure 1.** Visualization of cortical maps from 1 to 7 connectivity steps for the *local* (**a**) and *distributed* (**b**) connectivity using a single visual seed in the primary visual area of the occipital cortex in the *Emotion* task.

**
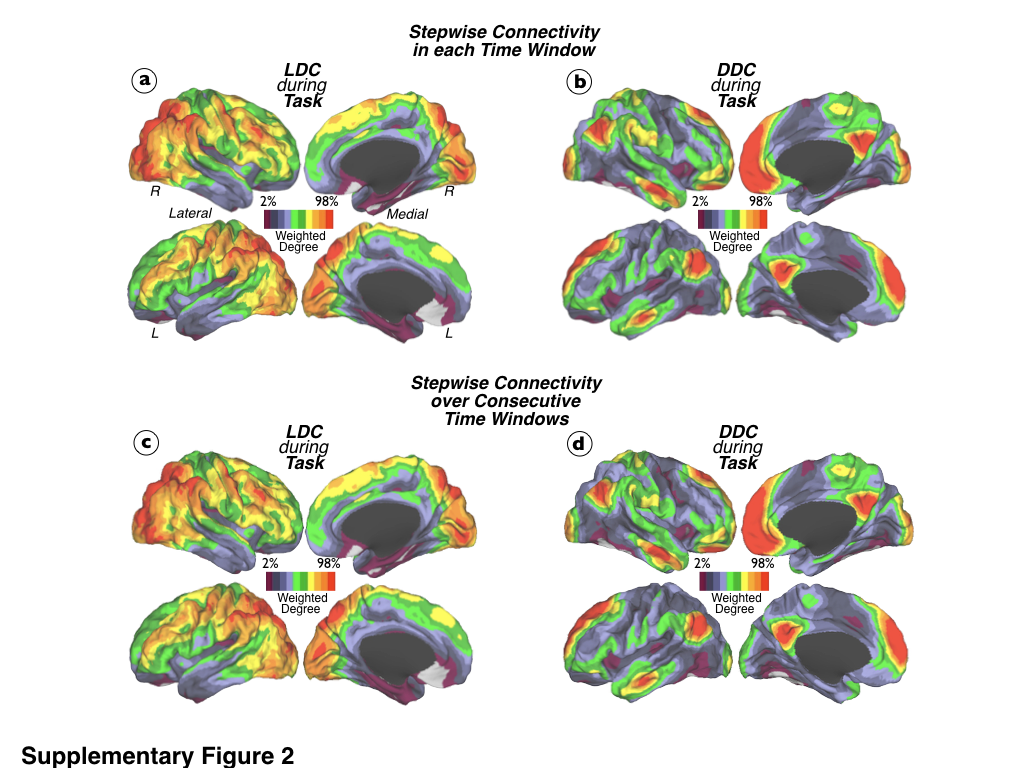
**Supplementary **Figure 2.** Calculation of SFC using discrete (**a** and **b**; 1 to 7 connectivity steps within the same association matrix of a given window; same as **Fig. 3**) or consecutive sliding windows (**c** and **d**; 1 to 7 connectivity steps transversally in consecutive association matrices) in the *local* and *distributed* connectivity conditions. Cortical maps show the average results for all cognitive tasks.

**
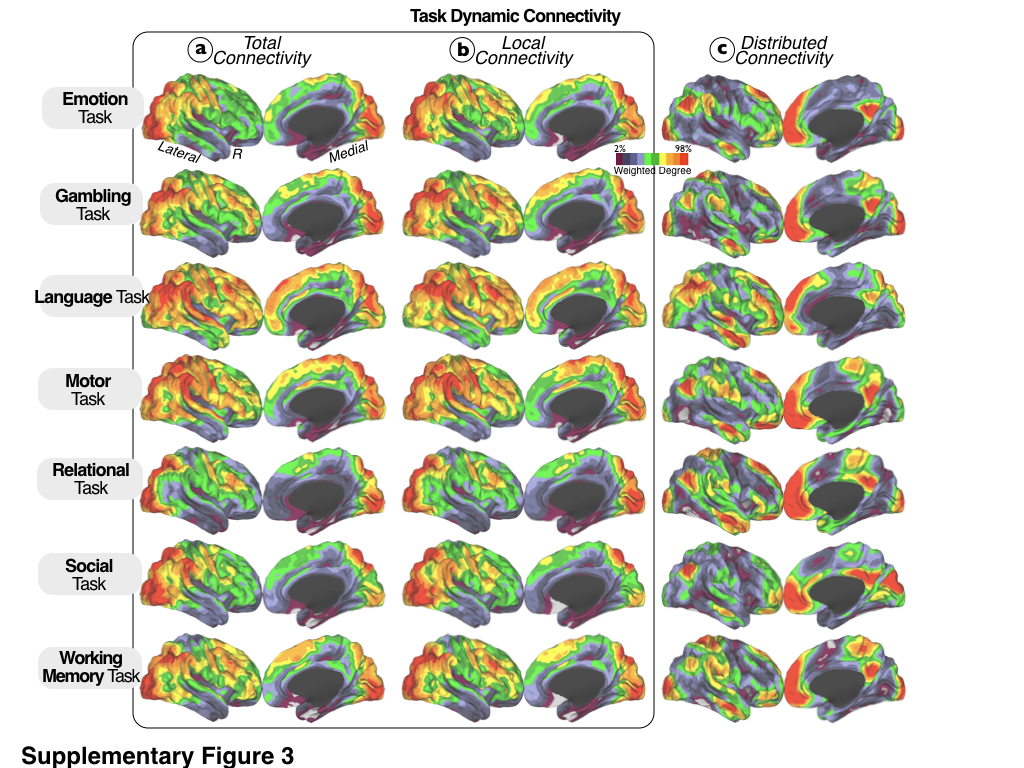
**Supplementary **Figure 3.** SFC analyses of the same data but including total dynamic connectivity (**a**), only *local* dynamic connectivity (**b**) or only *distributed* dynamic connectivity (**c**) in the 7 cognitive tasks.

**Supplementary Figure 4.** Customized high-pass cut-off filtering based on specific window sizes: 30, 45, 50 and 60 seconds.


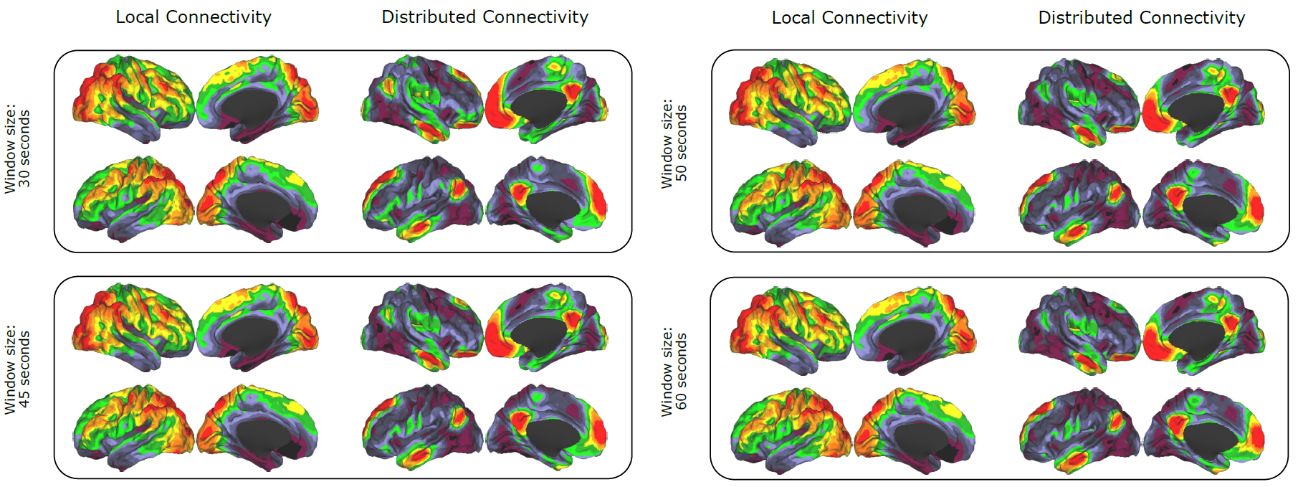

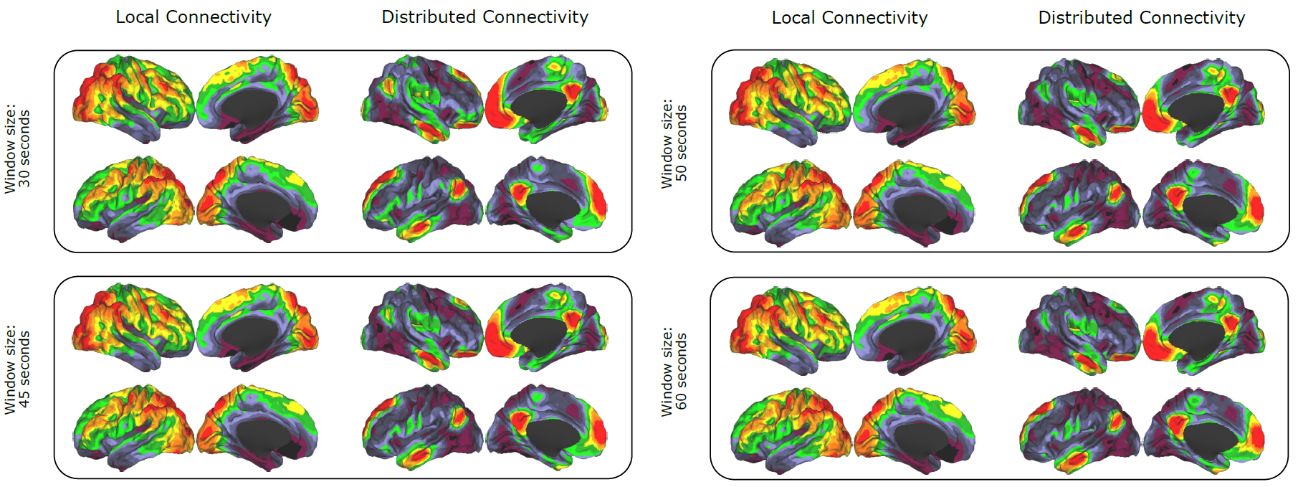


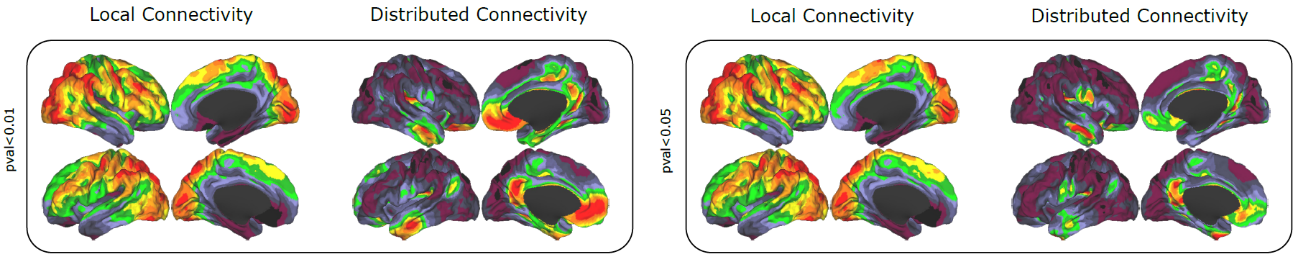


**Supplementary Figure 5.** Average maps of local and distributed connectivity patterns in “static” conditions (no sliding window approach).

**Supplementary Figure 6.** Local and distributed dynamic connectivity patterns of replication datasets 1 and 2. Cortical maps show the average of all task domains analogous to **Figure 3**.


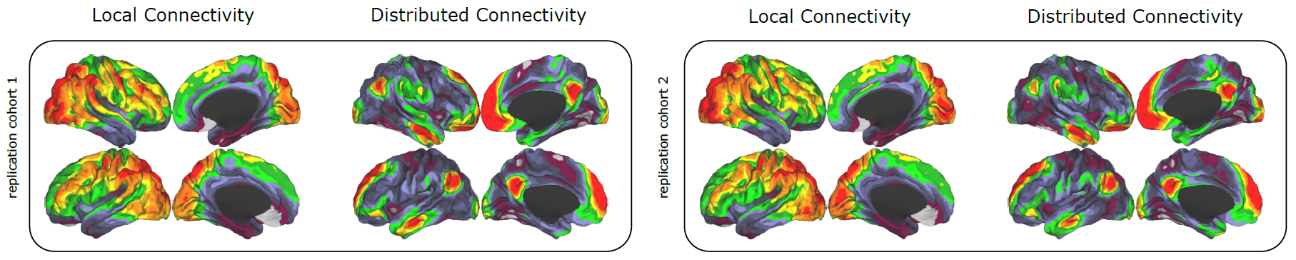

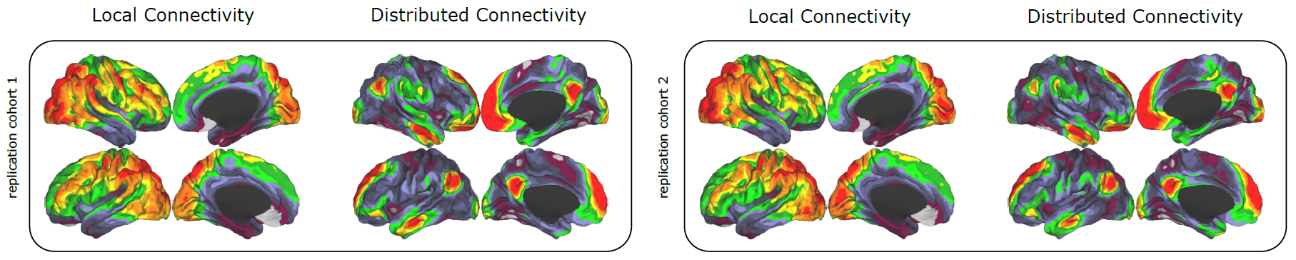


Supplementary Tables:

Supplementary **Table 1.** Imaging volumes and duration of 7 cognitive tasks.

| Task | Volumes | Duration (min:sec) |
| --- | --- | --- |
| Emotion Processing | 176 | 2:16 |
| Gambling | 253 | 3:12 |
| Language | 316 | 3:57 |
| Motor | 284 | 3:34 |
| Relational Processing | 232 | 2:56 |
| Social Cognition | 274 | 3:27 |
| Working Memory | 405 | 5:01 |

Supplementary Table 2. Customized high-pass cut-off filtering based on window size ($f_{min}=\frac{1}{w}$).

| Window Size  (seconds) | Number of time points in window | High pass filter cut-off frequency |
| --- | --- | --- |
| 30 | 42 | 0.033 |
| 45 | 63 | 0.022 |
| 50 | 70 | 0.02 |
| 60 | 84 | 0.016 |

**Supplementary Table 3.** Gene Ontology over-representation analysis of genes associated with the *local* dynamic connectivity map.

| **GO biological process complete** | **Fold Enrichment** | **P value uncorr** | **P value**  **FDR-corr** |
| --- | --- | --- | --- |
| Calcium ion-regulated exocytosis of neurotransmitter | 21.73 | 8.26E-09 | 4.35E-05 |
| Transmission of nerve impulse | 19.98 | 4.75E-10 | 1.15E-06 |
| Regulation of calcium ion-dependent exocytosis | 17.41 | 4.74E-11 | 7.70E-08 |
| Neuron-neuron synaptic transmission | 14.87 | 4.41E-08 | 7.84E-04 |
| Potassium ion transmembrane transport | 10.66 | 9.51E-11 | 1.80E-07 |
| Action potential | 10.38 | 1.21E-07 | 5.37E-04 |
| Regulation of postsynaptic membrane potential | 9.93 | 3.41E-04 | 3.57E-03 |
| Calcium ion transmembrane transport | 9.8 | 8.74E-10 | 1.31E-07 |
| Positive regulation of neuron projection development | 5.91 | 1.86E-06 | 3.69E-03 |
| Axonogenesis | 4.64 | 4.00E-06 | 4.19E-03 |
| Sensory perception | 3.63 | 6.66E-10 | 2.66E-06 |

**Supplementary Table 4.** Gene Ontology over-representation analysis of genes associated with the *distributed* dynamic connectivity map.

| **GO biological process complete** | **Fold Enrichment** | **P value uncorr** | **P value**  **FDR-corr** |
| --- | --- | --- | --- |
| Positive regulation of synapse assembly | 14.71 | 2.37E-07 | 8.42E-04 |
| Forebrain generation of neurons | 13.41 | 2.64E-07 | 1.69E-03 |
| Limbic system development | 12.06 | 5.81E-09 | 2.52E-05 |
| Neurotransmitter secretion | 12 | 1.28E-08 | 5.02E-06 |
| Cerebral cortex development | 10.09 | 1.27E-07 | 6.90E-04 |
| Positive regulation of synaptic transmission | 9.77 | 6.47E-09 | 4.03E-03 |
| Sensory perception | 8.83 | 1.54E-07 | 5.82E-04 |
| Axon guidance | 7.12 | 4.84E-08 | 4.03E-05 |
| Negative regulation of neurogenesis | 5.88 | 6.28E-06 | 3.82E-03 |

**Supplementary Table 5.** Gene Ontology over-representation analysis of genes associated with the *local* dynamic connectivity map without the a priori selection of neuro-related genes.

| **GO biological process complete** | **Fold Enrichment** | **P value uncorr** | **P value**  **FDR-corr** |
| --- | --- | --- | --- |
| Potassium ion transmembrane transport | 3.59 | 5.16E-08 | 6.20E-05 |
| Multicellular organismal signaling | 3 | 6.37E-05 | 3.55E-02 |
| Regulation of membrane potential | 2.11 | 9.02E-06 | 7.04E-03 |
| Regulation of ion transmembrane transport | 2.08 | 1.62E-05 | 1.10E-02 |
| Nervous system development | 1.51 | 1.22E-07 | 1.27E-04 |
| Regulation of cellular process | 1.14 | 6.58E-06 | 5.40E-03 |

**Supplementary Table 6.** Gene Ontology over-representation analysis of genes associated with the *distributed* dynamic connectivity map without the a priori selection of neuro-related genes.

| **GO biological process complete** | **Fold Enrichment** | **P value uncorr** | **P value**  **FDR-corr** |
| --- | --- | --- | --- |
| MAPK cascade | 2.01 | 1.86E-04 | 4.68E-02 |
| Transcription, DNA-templated | 1.56 | 8.22E-10 | 6.41E-06 |
| Regulation of transcription by RNA polymerase II | 1.49 | 4.07E-08 | 9.06E-05 |
| Nervous system development | 1.48 | 9.00E-07 | 5.02E-04 |

Supplementary Methods

*MRI data acquisition*

High-resolution anatomical MRI was acquired using a T1-weighted 3D MPRAGE sequence with the following parameters: TR = 2400 ms; TE = 2.14 ms; TI = 1000 ms; Flip angle = 8 deg; FOV = 224 x 224; Voxel size = isotropic 0.7 mm; BW = 210 (Hz/Px); iPAT: 2; Acquisition time 7 minutes and 40 seconds. To measure changes in blood-oxygenation-level-dependent (BOLD) T2* signals, during 7 different tasks that the subjects were asked to perform, a gradient-echo EPI sequence was used*. *The acquisitions were performed with the following parameters: TR = 720 ms, TE = 33.1 ms; Flip Angle 52; field of view 208x180 mm (RO x PE); 104 × 90 (RO x PE) matrix;72 slices with 2.0 mm isotropic voxels; multiband factor 8; echo spacing 0.58ms; and BW 2290 Hz/Px.

Subjects were asked to complete 7 different tasks inside the MR.

*Emotion processing task*

This task was adapted from the one developed by Hariri and colleagues (Hariri et al. 2002). Participants are presented with blocks of trials that either ask them to decide which of two faces presented on the bottom of the screen match the face at the top of the screen, or which of two shapes presented at the bottom of the screen match the shape at the top of the screen. The faces have either an angry or fearful expression. Trials are presented in blocks of 6 trials of the same task (face or shape), with the stimulus presented for 2000 ms and a 1000 ms ITI. Each block is preceded by a 3000 ms task cue (“shape” or “face”), so that each block is 21 seconds including the cue. Each of the two runs includes 3 face blocks and 3 shape blocks, with 8 seconds of fixation at the end of each run.

*Gambling task*

This task was adapted from the one developed by Delgado and Fiez (Delgado et al. 2000). Participants play a card guessing game where they are asked to guess the number on a mystery card (represented by a “?”) in order to win or lose money. Participants are told that potential card numbers range from 1-9 and to indicate if they think the mystery card number is more or less than 5 by pressing one of two buttons on the response box. Feedback is the number on the card (generated by the program as a function of whether the trial was a reward, loss or neutral trial) and either: 1) a green up arrow with “$1” for reward trials, 2) a red down arrow next to -$0.50 for loss trials; or 3) the number 5 and a gray double headed arrow for neutral trials. The “?” is presented for up to 1500 ms (if the participant responds before 1500ms, a fixation cross is displayed for the remaining time), following by feedback for 1000 ms. There is a 1000 ms ITI with a “+” presented on the screen. The task is presented in blocks of 8 trials that are either mostly reward (6 reward trials pseudo randomly interleaved with either 1 neutral and 1 loss trial, 2 neutral trials, or 2 loss trials) or mostly loss (6 loss trials pseudorandomly interleaved with either 1 neutral and 1 reward trial, 2 neutral trials, or 2 reward trials). In each of the two runs, there are 2 mostly reward and 2 mostly loss blocks, interleaved with 4 fixation blocks (15 seconds each).

*Language task*

This task was developed by Binder and colleagues (Binder et al. 2011) and uses the E-prime scripts provided by these investigators. The task consists of two runs that each interleave 4 blocks of a story task and 4 blocks of a math task. The lengths of the blocks vary (average of approximately 30 seconds), but the task was designed so that the math task blocks match the length of the story task blocks, with some additional math trials at the end of the task to complete the 3.8 minute run as needed. The story blocks present participants with brief auditory stories (5-9 sentences) adapted from Aesop’s fables, followed by a 2-alternative forced choice question that asks participants about the topic of the story. The example provided in the original Binder paper (p. 1466) is “For example, after a story about an eagle that saves a man who had done him a favor, participants were asked, “Was that about revenge or reciprocity?” The math task also presents trials aurally and requires subjects to complete addition and subtraction problems. The trials present subjects with a series of arithmetic operations (e.g., “fourteen plus twelve”), followed by “equals” and then two choices (e.g., “twenty-nine or twentysix”). Participants push a button to select either the first or the second answer. The math task is adaptive to try to maintain a similar level of difficulty across participants. For more details on the task, please see (Binder et al. 2011).

*Motor task*

This task was adapted from the one developed by Buckner and colleagues (Buckner et al. 2011; Yeo et al. 2011). Participants are presented with visual cues that ask them to either tap their left or right fingers, or squeeze their left or right toes, or move their tongue to map motor areas. Each block of a movement type lasted 12 seconds (10 movements), and is preceded by a 3 second cue. In each of the two runs, there are 13 blocks, with 2 of tongue movements, 4 of hand movements (2 right and 2 left), and 4 of foot movements (2 right and 2 left). In addition, there are 3 15-second fixation blocks per run.

*Relational processing task*

This task was adapted from the one developed by Christoff and colleagues (Smith et al. 2007). The stimuli are 6 different shapes filled with 1 of 6 different textures. In the relational processing condition, participants are presented with 2 pairs of objects, with one pair at the top of the screen and the other pair at the bottom of the screen. They are told that they should first decide what dimension differs across the top pair of objects (differed in shape or differed in texture) and then they should decide whether the bottom pair of objects also differ along that same dimension (e.g., if the top pair differs in shape, does the bottom pair also differ in shape). In the control matching condition, participants are shown two objects at the top of the screen and one object at the bottom of the screen, and a word in the middle of the screen (either “shape” or “texture”). They are told to decide whether the bottom object matches either of the top two objects on that dimension (e.g., if the word is “shape”, is the bottom object the same shape as either of the top two objects. For both conditions, the subject responds yes or no using one button or another. For the relational condition, the stimuli are presented for 3500 ms, with a 500 ms ITI, and there are four trials per block. In the matching condition, stimuli are presented for 2800 ms, with a 400 ms ITI, and there are 5 trials per block. Each type of block (relational or matching) lasts a total of 18 seconds. In each of the two runs of this task, there are 3 relational blocks, 3 matching blocks and 3 16-second fixation blocks.

*Social cognition task*

Participants were presented with short video clips (20 seconds) of objects (squares, circles, triangles) that either interacted in some way, or moved randomly on the screen. These videos were developed by either Castelli and colleagues (Castelli et al. 2000) or Martin and colleagues (Wheatley et al. 2007). After each video clip, participants judge whether the objects had a mental interaction (an interaction that appears as if the shapes are taking into account each other’s feelings and thoughts), Not Sure, or No interaction (i.e., there is no obvious interaction between the shapes and the movement appears random). Each of the two task runs has 5 video blocks (2 Mental and 3 Random in one run, 3 Mental and 2 Random in the other run) and 5 fixation blocks (15 seconds each).

*Working* *memory task*

The category specific representation task and the working memory task are combined into a single task paradigm. Participants were presented with blocks of trials that consisted of pictures of places, tools, faces and body parts (non-mutilated parts of bodies with no “nudity”). Within each run, the 4 different stimulus types were presented in separate blocks. Also, within each run, ½ of the blocks use a 2-back working memory task and ½ use a 0-back working memory task (as a working memory comparison). A 2.5 second cue indicates the task type (and target for 0-back) at the start of the block. Each of the two runs contains 8 task blocks (10 trials of 2.5 seconds each, for 25 seconds) and 4 fixation blocks (15 seconds). On each trial, the stimulus is presented for 2 seconds, followed by a 500 ms inter-task interval (ITI).

Data preprocessing

The fMRI data was pre-processed using FSL and AFNI. In the first place, the fMRI dataset was aligned to the middle volume, using a six parameter (rigid body) linear transformation, to correct for head movement artefacts; the transformation matrix of each volume to the middle volume was used to compute 24 motion parameters. After intensity normalization, the 24 motion parameters, the average cerebrospinal fluid (CSF) signal and the average white-matter signal were regressed out, followed by the removal of linear and quadratic trends. Next the functional data was spatially normalized to the MNI152 brain template, with a voxel size of 3*3*3 mm3 and smoothed with a 6 mm full width at half maximum (FWHM) isotropic Gaussian kernel. Finally, a down-sampling to 8 mm was applied to compute graph analysis method at the voxel level.
